# Improving the Current Spreading by Locally Modulating the Doping Type in the n-AlGaN Layer for AlGaN-Based Deep Ultraviolet Light-Emitting Diodes

**DOI:** 10.1186/s11671-019-3078-8

**Published:** 2019-08-06

**Authors:** Jiamang Che, Hua Shao, Jianquan Kou, Kangkai Tian, Chunshuang Chu, Xu Hou, Yonghui Zhang, Qian Sun, Zi-Hui Zhang

**Affiliations:** 1 State Key Laboratory of Reliability and Intelligence of Electrical Equipment, 5340 Xiping Road, Beichen District, Tianjin, 300401 People’s Republic of China; 2 Key Laboratory of Electronic Materials and Devices of Tianjin, School of Electronics and Information Engineering, Hebei University of Technology, 5340 Xiping Road, Beichen District, Tianjin, 300401 People’s Republic of China; 3 Key Laboratory of Nanodevices and Applications, Suzhou Institute of Nano-Tech and Nano-Bionics (SINANO), Chinese Academy of Sciences (CAS), Suzhou, 215123 People’s Republic of China

**Keywords:** DUV LED, Current spreading effect, Conduction band barrier height, External quantum efficiency, Wall-plug efficiency

## Abstract

In this report, we locally modulate the doping type in the *n*-AlGaN layer by proposing n-AlGaN/p-AlGaN/n-AlGaN (NPN-AlGaN)-structured current spreading layer for AlGaN-based deep ultraviolet light-emitting diodes (DUV LEDs). After inserting a thin p-AlGaN layer into the n-AlGaN electron supplier layer, a conduction band barrier can be generated in the *n*-type electron supplier layer, which enables the modulation of the lateral current distribution in the p-type hole supplier layer for DUV LEDs. Additionally, according to our studies, the Mg doping concentration, the thickness, the AlN composition for the p-AlGaN insertion layer and the NPN-AlGaN junction number are found to have a great influence on the current spreading effect. A properly designed NPN-AlGaN current spreading layer can improve the optical output power, external quantum efficiency (EQE), and the wall-plug efficiency (WPE) for DUV LEDs.

## Introduction

Owing to various applications such as disinfection, water purification, medical treatment, and high-density optical recording [1–8], intensive efforts have been invested for developing high-efficiency AlGaN-based deep ultraviolet light-emitting diodes (DUV LEDs). At the current stage, remarkable progress has been achieved to improve the crystalline quality for Al-rich AlGaN films, e.g., growing AlN films on nano-patterned sapphire substrates by graphene-assisted quasi-Van der Waals epitaxy can greatly release the strain and reduce the dislocation density [9], which indicates the internal quantum efficiency (IQE) of 80% [10]. It is worth noting that such IQE is measured by using the low-temperature photoluminescence method, which does not get any carrier injection involved. However, DUV LEDs are operated by electrical bias, which is associated with current flow and carrier transport [11–13]. Another very important aspect regarding the current flow is the current crowding effect, which easily takes place when the device is biased at a very high current level [14]. DUV LEDs have a very inferior Mg doping efficiency in the p-AlGaN layer with high AlN component [15, 16], leading to low electrical conductivity. Moreover, DUV LEDs adopt the flip-chip structures that feature the lateral injection scheme for the current. Hence, compared to InGaN/GaN-based UV, blue and green LEDs, AlGaN-based DUV LEDs are more challenged by the current crowding effect [17]. The occurrence of the current crowding effect either at the p-contact electrode or at the mesa edge leads to uneven electroluminescence intensity in the multiple quantum wells (MQWs) and the increased junction temperature [18]. As a result, it is indeed crucial to promote the lateral current spreading for DUV LEDs. For that purpose, the proposed narrow-multiple-strip p-type electrode enables an evenly distributed current spreading, thus increasing the wall-plug efficiency (WPE) by 60% [19]. Moreover, ITO/ZGO (ZnGaO) current spreading layer can better spread the current and improve the external quantum efficiency (EQE), but the increased interfacial resistivity at the ZGO/p-GaN interfaces makes the WPE less enhanced for DUV LEDs [20].

Therefore, at the current stage, research attention is laid on the p-side to facilitate the current spreading for DUV LEDs. In this work, different than other approaches, we propose and prove that the improved current distribution in the p-type hole supplier layer for DUV LEDs can be achieved by engineering the n-AlGaN electron supplier layer. The energy barrier is generated in the conduction band by modulating the doping type in the electron supplier layer, i.e., the n-AlGaN/p-AlGaN/n-AlGaN (NPN-AlGaN) structure is proposed and parametrically studied. Our results show that the lateral distribution for the holes can be homogenized by using the NPN-AlGaN junction, which therefore enhances the optical output power, the external quantum efficiency, and the wall-plug efficiency for DUV LEDs. Another advantage of our design is that, from the point of view of epitaxial growth, having the current spreading layer in the n-type electron supplier layer allows the epi-growers more freedom in optimizing the growth conditions.

## Research Methods and Physics Models

**Fig. 1:**
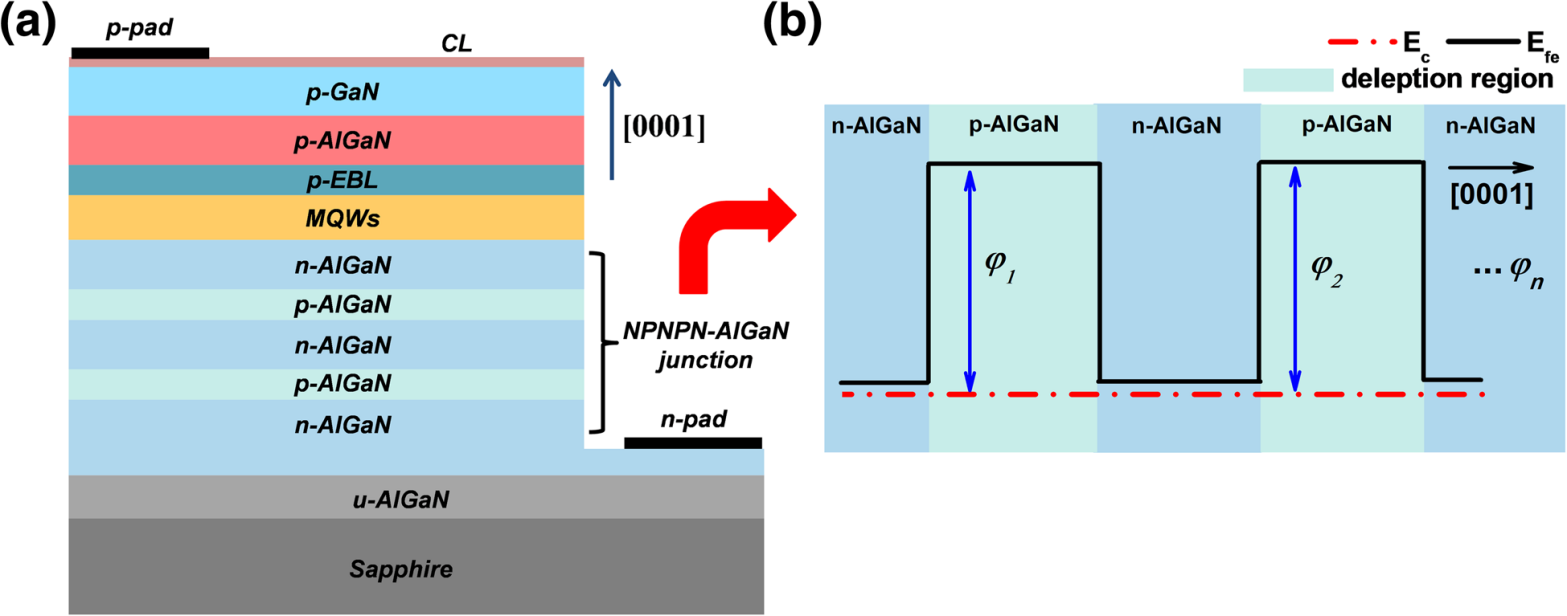
**a** Schematic structures for the NPN-AlGaN LED. **b** Schematic conduction band profile for the NPNPN-AlGaN structure having two NPN-AlGaN junctions; we define the barrier heights for each NPN-AlGaN junction as *φ*_1_, *φ*_2_, and *φ*_*n*_, and *n* is the number of NPN-AlGaN junction

The NPN-AlGaN DUV LED structures are schematically drawn in Fig. 1a. In each studied DUV LED, we have a 4-μm-thick n-Al_0.60_Ga_0.40_N/p-Al_*x*_Ga_1−*x*_N/n-Al_0.60_Ga_0.40_N layer, and the Si doping concentration of the n-Al_0.60_Ga_0.40_N region is 5 × 10^18^ cm^−3^. Then, five pairs of Al_0.45_Ga_0.55_N/Al_0.56_Ga_0.44_N multiple quantum well (MQW) active layers are designed, for which the thicknesses of quantum wells and quantum barriers are 3 nm and 12 nm, respectively. The MQWs are capped by an 18-nm-thick Mg-doped p-Al_0.60_Ga_0.40_N layer serving as the p-EBL, after which a 50-nm-thick Mg-doped p-Al_0.40_Ga_0.60_N layer and a 50-nm-thick Mg-doped p-GaN layer follow. The hole concentration for the p-EBL and the hole supplier layers are set to be 3 × 10^17^ cm^−3^. We design the device geometry with a rectangular mesa of 350 × 350 μm^2^. Figure 1b shows the schematic conduction band profiles when two NPN-AlGaN junctions (i.e., NPNPN-AlGaN structure) are employed in the DUV LED structure, and we can see the energy barriers existing in the depleted p-Al_*x*_Ga_1−*x*_N regions. The energy barriers can adjust the horizontal current distribution in the p-type hole supplier layer. Note, to guarantee the current stream through the reversely biased n-AlGaN/p-AlGaN junction, it is very important to have the p-AGaN insertion layer fully depleted so that the NPN-AlGaN junction will be in a reach-through breakdown mode [21]. Detailed analysis and discussions will be presented subsequently. Our reference DUV LED is identical to the NPN-AlGaN DUV LEDs except that the 4-μm-thick Si-doped n-Al_0.60_Ga_0.40_N layer is utilized as the electron supplier layer.

**Fig. 2:**
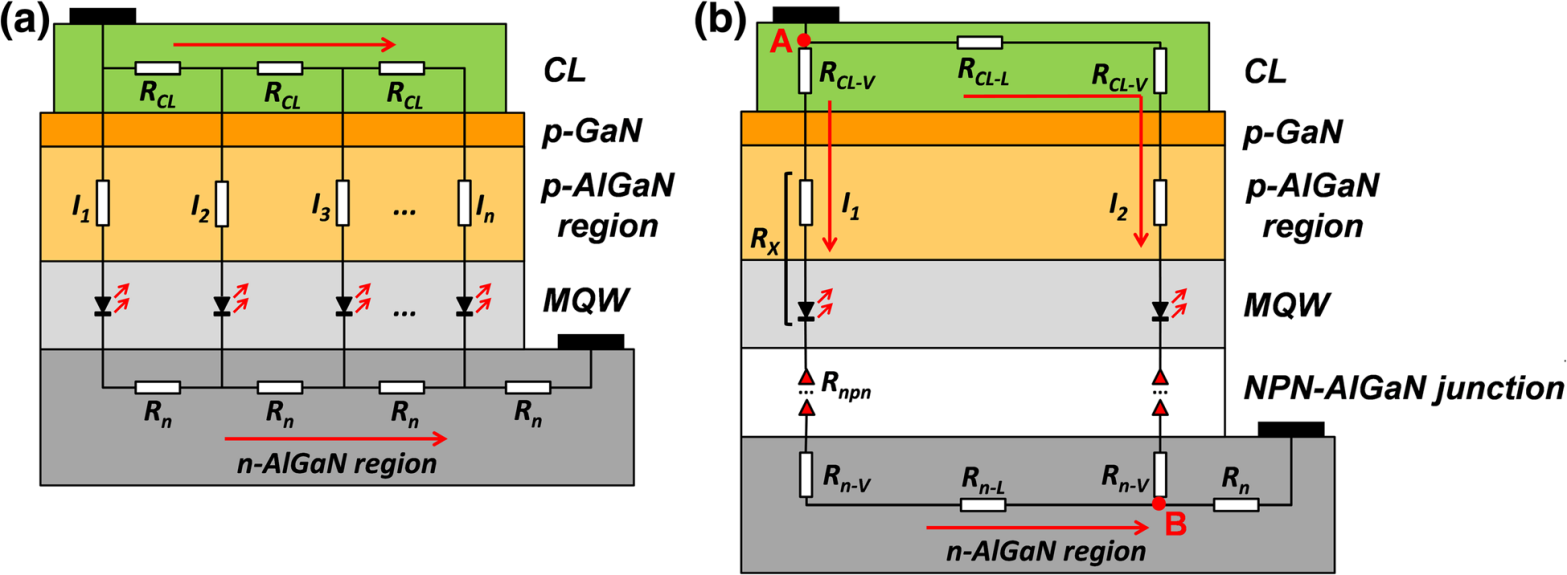
**a** DUV LEDs with lateral current-injection scheme equivalent circuit (*I*_1_ > *I*_2_ > *I*_3_ > …… > *I*_*n*_). **b** NPN-AlGaN-structured DUV LED simplified equivalent circuit and current paths *I*_1_ and *I*_2_ are exhibited

To better understand the physical mechanism for the improved current spreading effect that is enabled by the NPN-AlGaN junction, an equivalent circuit for the DUV LED with a lateral current-injection scheme is shown in Fig. 2a. We can see that the current flows from the p-type hole supplier layer to the n-AlGaN region along both vertical and lateral directions. If the electrical resistance for the n-AlGaN electron supplier layer is smaller than that for the current spreading layer (CL), the current tends to crowd in the region under the p-type ohmic contact, i.e., *I*_1_ > *I*_2_ > *I*_3_ > … > *I*_*n*_ [14]. The incorporation of NPN-AlGaN junctions in the DUV LED structure can suppress the destructive current crowding effect. Then, we further simplify the current flow paths for the NPN-AlGaN DUV LED in Fig. 2b, such that the total current can be divided into a vertical portion (*I*_1_) and a horizontal portion (*I*_2_) from point *A* to point *B*. Therefore, the total voltage between the two points is shared by the current spreading layer, the p-GaN layer, the p-AlGaN layer, the MQWs, the NPN-AlGaN junctions, and the n-AlGaN layer. Based on the current paths of *I*_1_ and *I*_2_, Eqs. 1 and 2 are obtained, respectively, and by solving the previous two formulas, Eq. 3 is then derived:

1 $$ {I}_1{R}_{\mathrm{CL}-\mathrm{V}}+{I}_1{R}_X+{I}_1\bullet N\bullet {R}_{npn}+{I}_1\left({R}_{n-\mathrm{V}}+{R}_{n-L}\right)={U}_{\mathrm{AB}}, $$

2 $$ {I}_2\left({R}_{\mathrm{CL}-\mathrm{L}}+{R}_{\mathrm{CL}-\mathrm{V}}\right)+{I}_2{R}_X+{I}_2\bullet N\bullet {R}_{npn}+{I}_2{R}_{n-\mathrm{V}}={U}_{\mathrm{AB}}, $$

3 $$ \frac{I_1}{I_2}=1+\frac{R_{\mathrm{CL}-\mathrm{L}}-{R}_{n-\mathrm{L}}}{R_{\mathrm{CL}-\mathrm{V}}+{R}_X+{R}_n+N\bullet {R}_{npn}} $$

where *R*_*CL−V*_ and *R*_*CL−L*_ are the vertical and horizontal resistances for the current spreading layer, respectively; *R*_*n*−*V*_ and *R*_*n*−*L*_ denote the vertical and horizontal resistances for the n-AlGaN layer, respectively; *R*_*n*_ is the summation of *R*_*n*−*V*_ and *R*_*n*−*L*_ (i.e., *R*_*n*_ = *R*_*n*−*V*_ + *R*_*n*−*L*_) for the current path *I*_1_; the summation of the resistance for the p-type hole injection region and MQW region is represented by *R*_*x*_; *R*_*npn*_ is the interfacial resistance induced by the barrier height in each NPN-AlGaN junction; *N* means the total number for the NPN-AlGaN junction, and the total voltage drop between points *A* and *B* is described by *U*_*AB*_. It is worth mentioning that the 200-nm-thick current spreading layer is much thinner than the 4-μm-thick n-AlGaN electron supplier layer for all studied devices. Therefore, a CL of which the electrical resistance is much bigger than that for n-AlGaN layer is obtained, i.e., *R*_*CL−L*_ − *R*_*n*−*L*_ ≫ 0. It is obvious that the ratio of *I*_1_/*I*_2_ can be reduced by making *N* × *R*_*npn*_ value increase*.* Hence, the current spreading effect in the p-type hole supplier layer can be improved by using the NPN-AlGaN junction in the n-type electron supplier layer for DUV LED structures. On one hand, the *N* × *R*_*npn*_ value can be enhanced through increasing *N*. On the other, the value of *R*_*npn*_ is affected by the AlN component, the thickness, and the Mg doping concentration for the p-AlGaN insertion layer. Therefore, a detailed analysis will be conducted in the subsequent discussions.

Crosslight APSYS simulator is used to investigate the device physics, and the models that we use are reliable according to our previous publications on blue, UVA, and DUV nitride-based LEDs [22–24]. In our physical models, the energy band offset ratio for the AlGaN/AlGaN heterojunction is set to be 50:50 [25]. The Auger recombination coefficient, the Shockley-Read-Hall (SRH) recombination lifetime, and the light extraction efficiency are set to be 1.0 × 10^−30^ cm^6^/s [26], 10 ns [27], and ~ 8% [28] for DUV LEDs, respectively. The polarization-induced interface charges at the lattice-mismatched interface are considered by assuming the polarization level of 40% [29].

## Results and Discussions

### Influence of the NPN-AlGaN Structure on the Current Spreading Effect for DUV LEDs

**Fig. 3:**
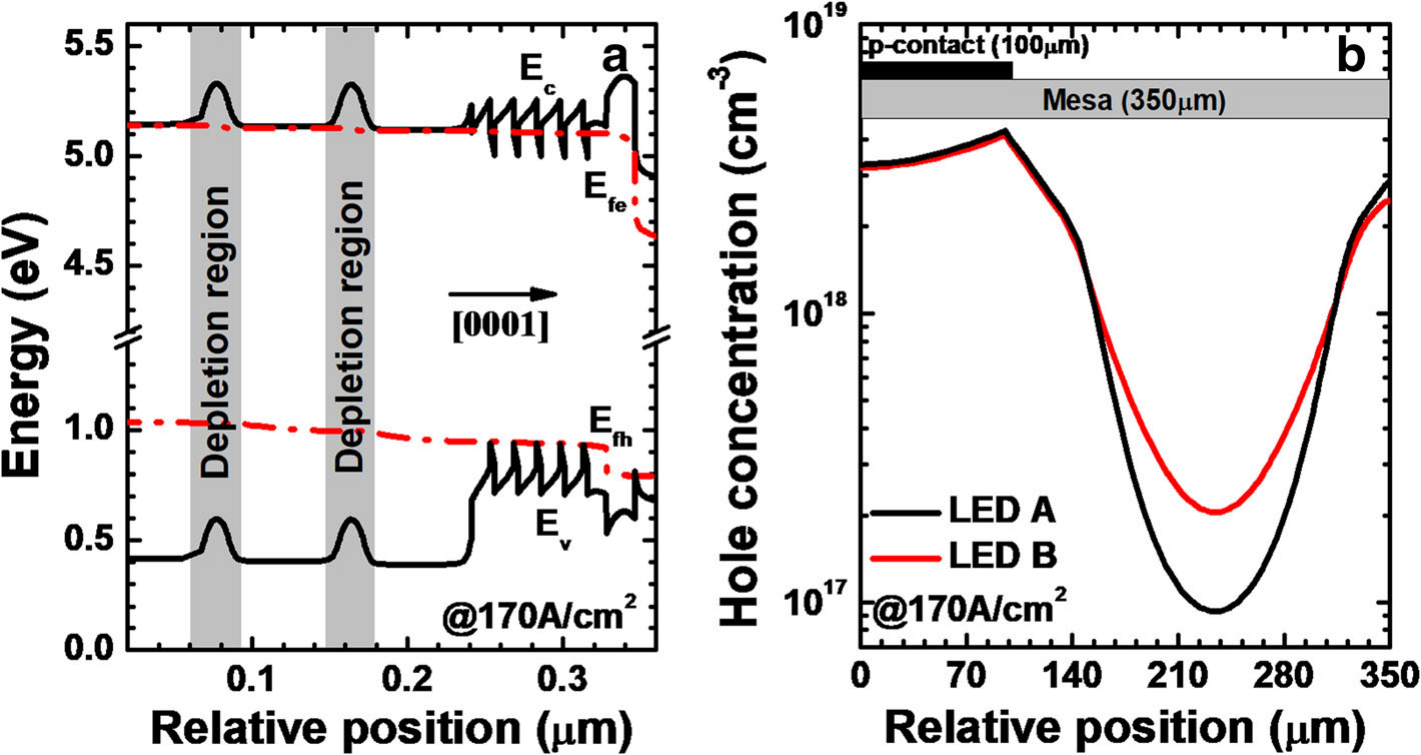
**a** Energy band profile for LED B, in which we define the conduction band, quasi-Fermi levels for electrons and holes, and the valence band as *E*_*c*_, *E*_*fe*_, *E*_*fh*_, and *E*_*v*_, respectively. **b** Horizontal hole concentration in the LQW for LEDs A and B when the current density is 170 A/cm^2^

LED A (i.e., the reference DUV LED without NPN-AlGaN junction) and LED B (i.e., the DUV LED with NPN-AlGaN junction) have been firstly investigated to probe the influence of the NPN-AlGaN structure in homogenizing the current for the p-type hole supplier layer. Each NPN-AlGaN junction has a 20-nm-thick p-Al_0.60_Ga_0.40_N insertion layer, for which the Mg doping concentration is 1 × 10^18^ cm^−3^. Figure 3a shows the energy band profile when the current density is 170 A/cm^2^ for LED B. Two energy barriers in the conduction band are formed in the NPN-AlGaN junctions, and the formation of the energy barrier is well ascribed to the depletion effect of the inserted p-Al_0.60_Ga_0.40_N layer. The generated barriers in LED B induce the interfacial resistance of *R*_*npn*_ in the NPN-AlGaN junction region, which helps decrease *I*_1_/*I*_2_ as mentioned in Eq. 3, such that more holes will flow along the current path *I*_2_. We then calculate and show the horizontal hole concentration in the last quantum well (LQW) for LEDs A and B when the current density is 170 A/cm^2^, as presented in Fig. 3b. We can clearly see that LED B obtains a better lateral current spreading when compared with LED A. Hence, we prove that the NPN-AlGaN in the n-type electron supplier layer facilitates the current spreading effect in the p-type hole supplier layer for DUV LEDs.

**Fig. 4:**
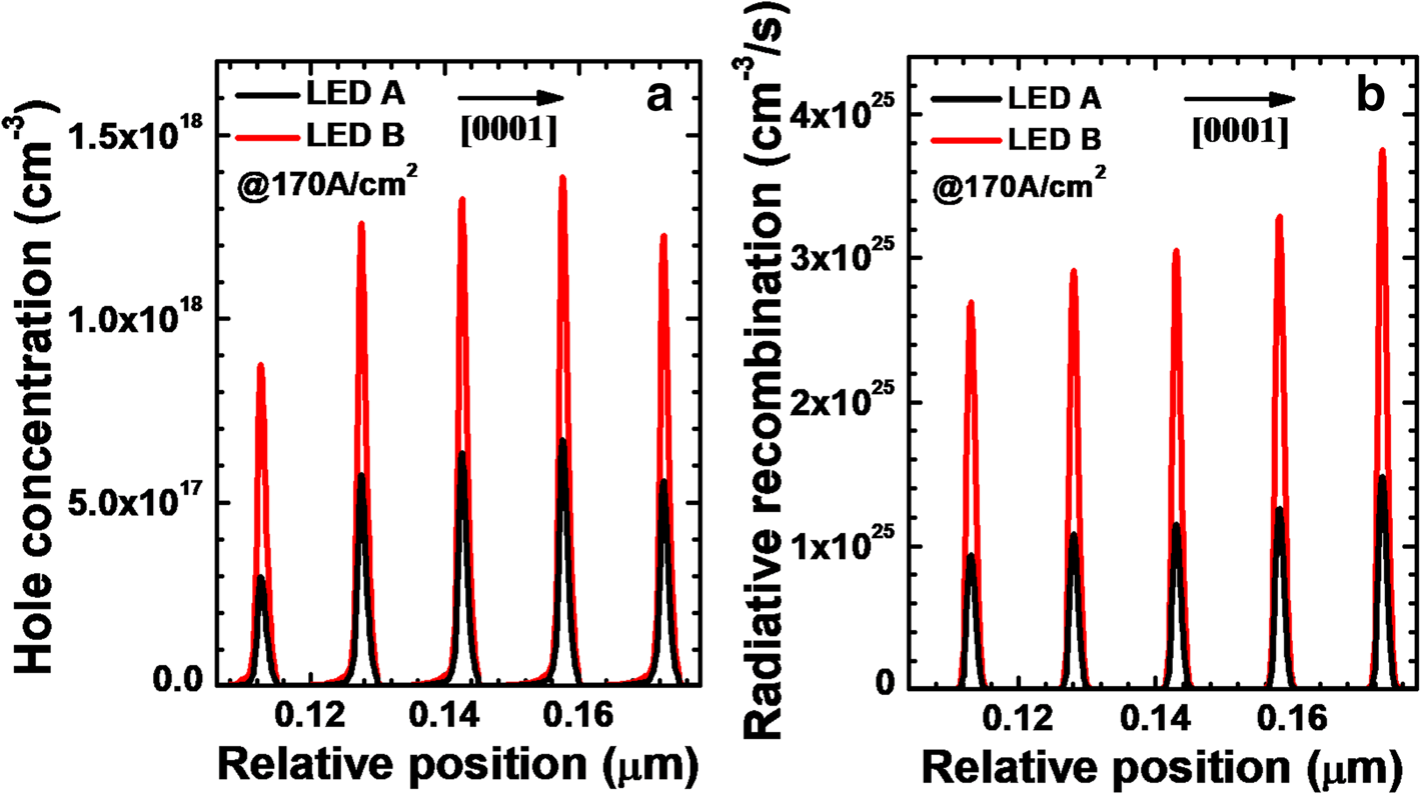
**a** Hole concentration levels and **b** radiative recombination profiles in the MQWs for LEDs A and B, respectively. We collect the data at the location of 120 μm away from the right edge of the mesa when the current density is 170 A/cm^2^

Besides showing the lateral hole concentration, we also demonstrate the hole concentration levels in the MQWs for LEDs A and B in Fig. 4a. We can see that, because of the improved current spreading effect, the hole concentration in the MQWs is enhanced for LED B when compared to that for LED A. The enhanced hole concentration level in the MQWs more favors the radiative recombination for LED B (see Fig. 4b).

**Fig. 5:**
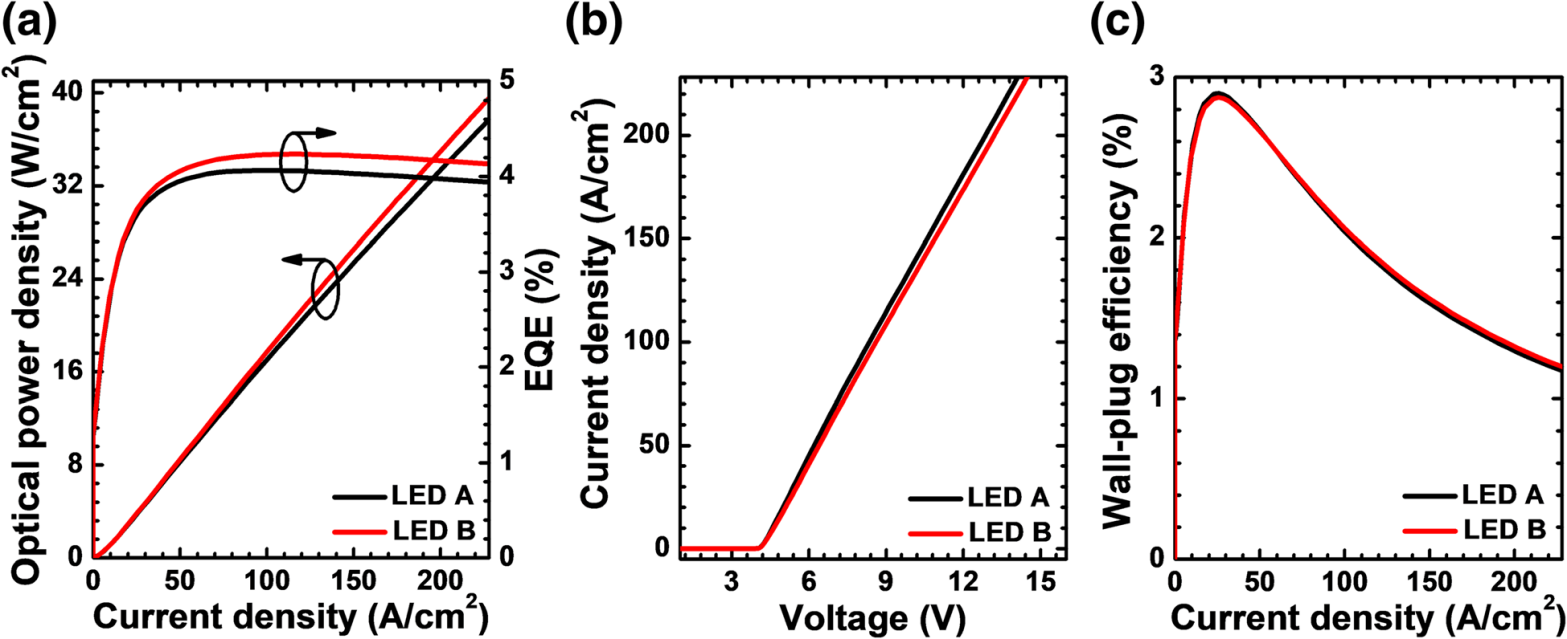
**a** EQE and optical power density in terms of the injection current, **b** current-voltage characteristic, and **c** WPE as a function of the injection current for LEDs A and B

The impact of the NPN-AlGaN junction is also justified by the calculated optical and electrical performances for LEDs A and B as shown in Fig. 5. Figure 5a presents the EQE and the optical power density as a function of the injected current for both LEDs A and B. We can see that LED B has both higher EQE and optical power density than LED A, thanks to the improved current spreading effect and hole injection efficiency enabled by the NPN-AlGaN junction. For instance, the optical power density enhancement for LED B is ~ 1.67% when the current density is 170 A/cm^2^ according to Fig. 5a. Investigations in Fig. 5b illustrate that the forward voltage for LED B with NPN-AlGaN junction has a slight increase when compared with that for LED A. We attribute this phenomenon to the energy barriers in the depletion regions that are caused by the NPN-AlGaN junctions. Fortunately, the higher forward voltage of LED B does not have a detrimental effect on wall-plug efficiency (WPE), and the WPE for LED B exceeds that for LED A when the injection current density is larger than ~ 56 A/cm^2^ as shown in Fig. 5c. We believe that both enhanced EQE and WPE can be realized once the NPN-AlGaN junction can be optimized, which will be fully investigated as follows.

### Effect of the AlN Composition for p-AlGaN Layer on the Current Spreading Effect

**Fig. 6:**
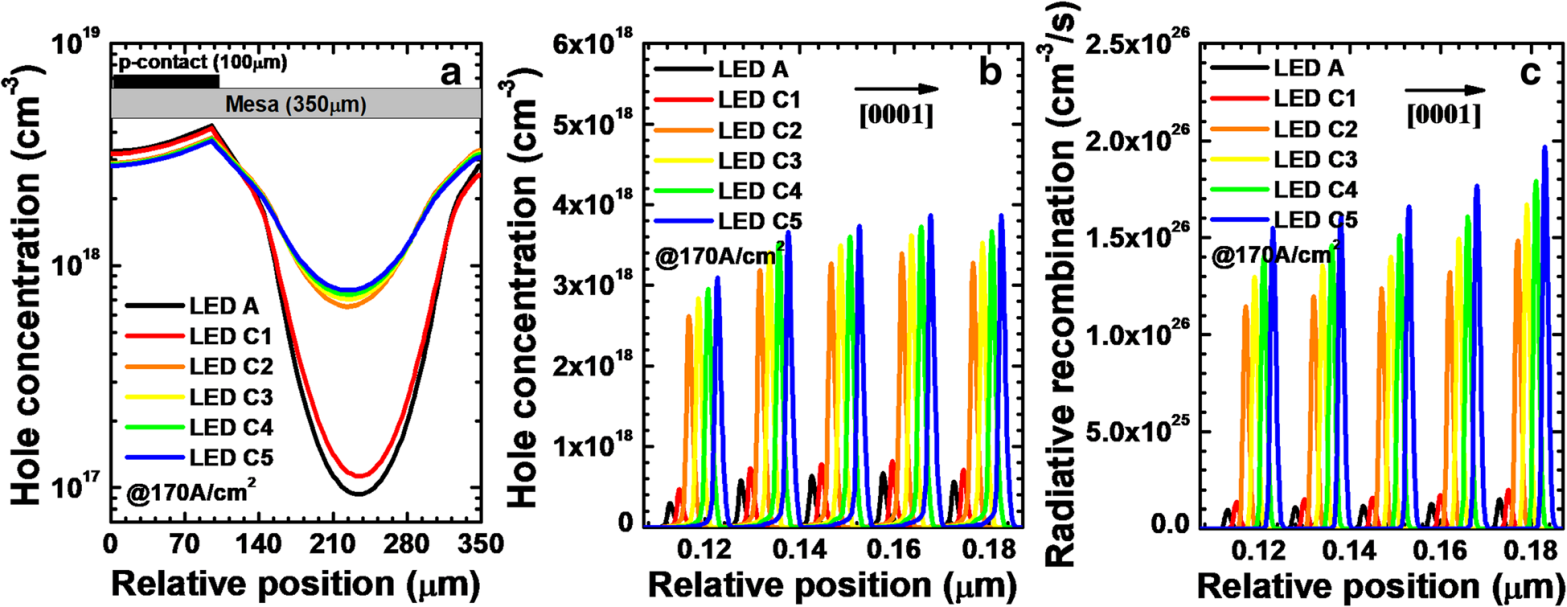
**a** Horizontal hole concentration in the LQW, **b** hole concentration levels, and **c** radiative recombination profiles in the MQWs for LEDs A and D*i* (*i* = 1, 2, 3, 4, and 5) when the current density is 170 A/cm^2^. We purposely shift the curves for **b** and **c** by 2 nm for easier identification

In this section, the impact of AlN composition for the NPN-AlGaN junction on the optical and electrical properties for DUV LEDs is studied. In order to clearly illustrate this mechanism, we use five DUV LEDs, i.e., LEDs C*i* (*i* = 1, 2, 3, 4, and 5) with different NPN-Al_*x*_Ga_1−*x*_N junctions, for which the AlN compositions for p-Al_*x*_Ga_1−*x*_N insertion layers are 0.60, 0.63, 0.66, 0.69, and 0.72, respectively. The doping concentration and thickness for the p-Al_*x*_Ga_1−*x*_N layer are 1.8 × 10^18^ cm^−3^ and 20 nm, respectively. Two NPN-AlGaN junctions, i.e., NPNPN-AlGaN junction are used for all studied devices. We then calculate the conduction band barrier height for each NPN-Al_*x*_Ga_1−*x*_N junction for LEDs C*i* (*i* = 1, 2, 3, 4, and 5) as shown in Table 1. It is distinct to see that the value of the conduction barrier height increases as the AlN composition for the p-Al_*x*_Ga_1−*x*_N insertion layer increases. High conduction barrier height can make the value of *R*_*npn*_ increase, and a decreased ratio of *I*_1_/*I*_2_ is simultaneously triggered as mentioned in Eq. 3. To prove that point, the lateral hole distributions in the last quantum well for all studied devices when the current density is 170 A/cm^2^ are calculated and exhibited in Fig. 6a. For LED C1, the hole distribution can be modulated after the NPN-Al_0.60_Ga_0.40_N structure is adopted, and it is obvious that the current spreading effect obtains further improvement once the AlN component of p-AlGaN insertion layer increases up to 0.63 for our structures.

**Table 1:** Conduction band barrier heights of each NPN-AlGaN junction for LEDs C*i* (*i* = 1, 2, 3, 4, and 5)

LEDs	C1	C2	C3	C4	C5
*φ*_1_ (eV)	0.230	0.252	0.254	0.257	0.258
*φ*_2_ (eV)	0.236	0.251	0.254	0.256	0.258

We demonstrate the simulated hole concentration levels and radiative recombination profiles in the MQWs for LEDs A and C*i* (*i* = 1, 2, 3, 4, and 5) in Fig. 6b and c when the current density is 170 A/cm^2^, respectively. The hole concentration levels and radiative recombination profiles are collected at the location of 120 μm away from the right edge of the mesa. We spatially shift the hole concentration levels and radiative recombination profiles in Fig. 6b and c for the investigated DUV LEDs by 2 nm for an easier identification, respectively. The lowest hole concentration in the MQWs is clearly observed for LED A, and thus, the lowest radiative recombination is also shown in Fig. 6c. The hole concentration and radiative recombination in the MQWs increase due to the adoption of NPN-AlGaN junction, and they can be even more increased with the increase of AlN composition in the p-AlGaN insertion layer.

**Fig. 7:**
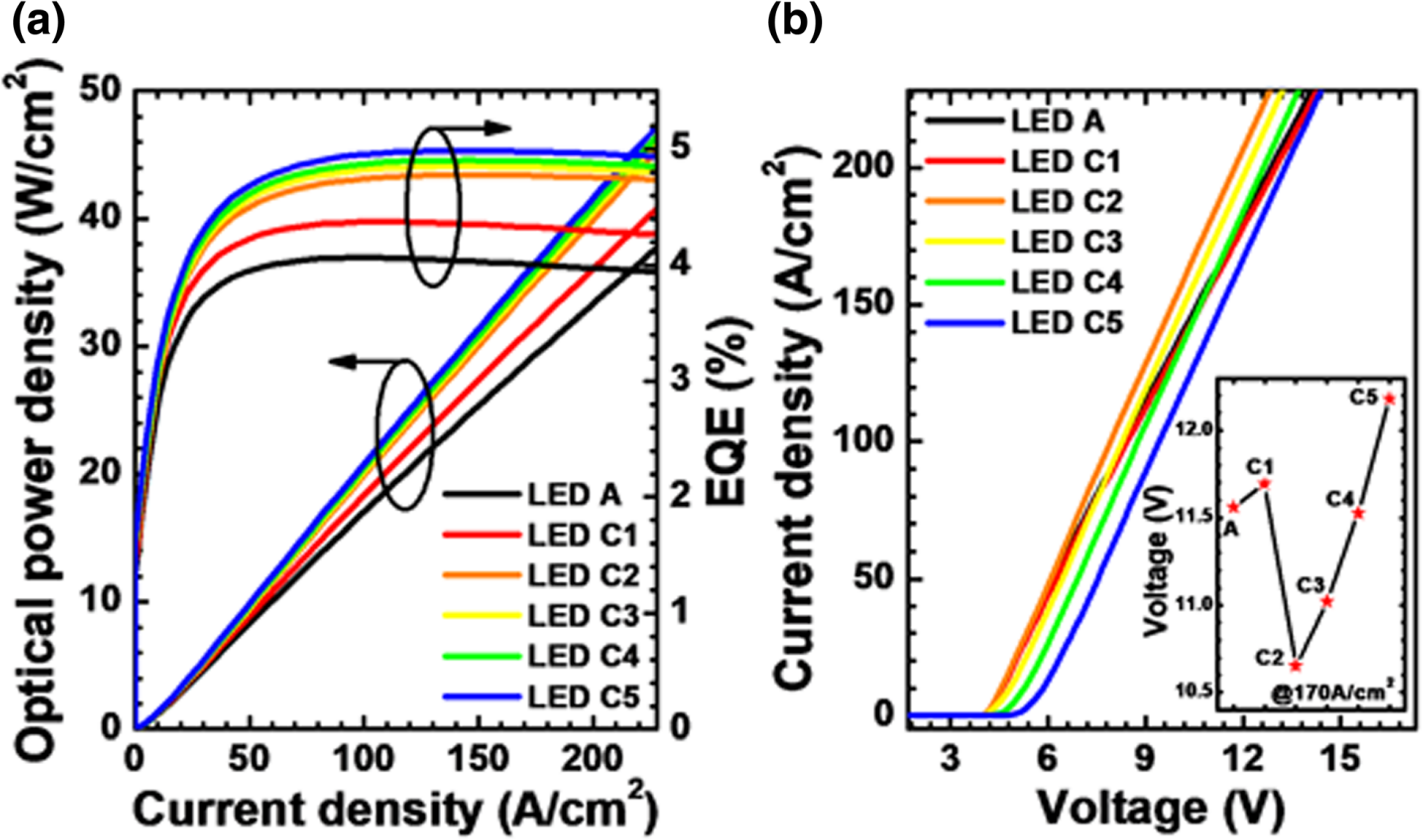
**a** EQE and optical power density in terms of the injection current and **b** current-voltage characteristics for LEDs A and C*i* (*i* = 1, 2, 3, 4, and 5). Inset: the forward voltages for LEDs A and C*i* (*i* = 1, 2, 3, 4, and 5) when the current density is 170 A/cm^2^

The optical power density and EQE as a function of the injection current density are further calculated and exhibited for the studied LEDs in Fig. 7a. As shown in the figure, the EQE and optical power density increase once the NPN-AlGaN junction is adopted. Moreover, the EQE and optical power density can be further promoted as the AlN composition for the p-AlGaN insertion layer increases. We contribute this to the more homogeneous lateral hole distribution in the MQWs as shown in Fig. 6a. The current-voltage characteristics for LEDs A and C*i* (*i* = 1, 2, 3, 4, and 5) are presented in Fig. 7b. The forward voltage for LED C1 shows a small increase when compared with LED A, and LED C5 shows the largest forward voltage. The inset figure shows the forward voltage for all studied LEDs when the current density is 170 A/cm^2^. It is noteworthy that the forward voltage decreases for LEDs C2, C3, and C4 when compared with LED A. Although the NPN-AlGaN junction increases the vertical resistance for DUV LEDs, the more uniform carrier concentration along the horizontal direction improves the horizontal conductivity, thus leading to a reduced forward voltage. It indicates that the enhanced current spreading effect can help to reduce the forward operating voltage for DUV LEDs as long as the current spreading layer is properly designed [30]. However, our design modulates the current path by inducing barriers, and hence, a too much high barrier height may sacrifice the electrical conductance [21], e.g., LED C5.

**Fig. 8:**
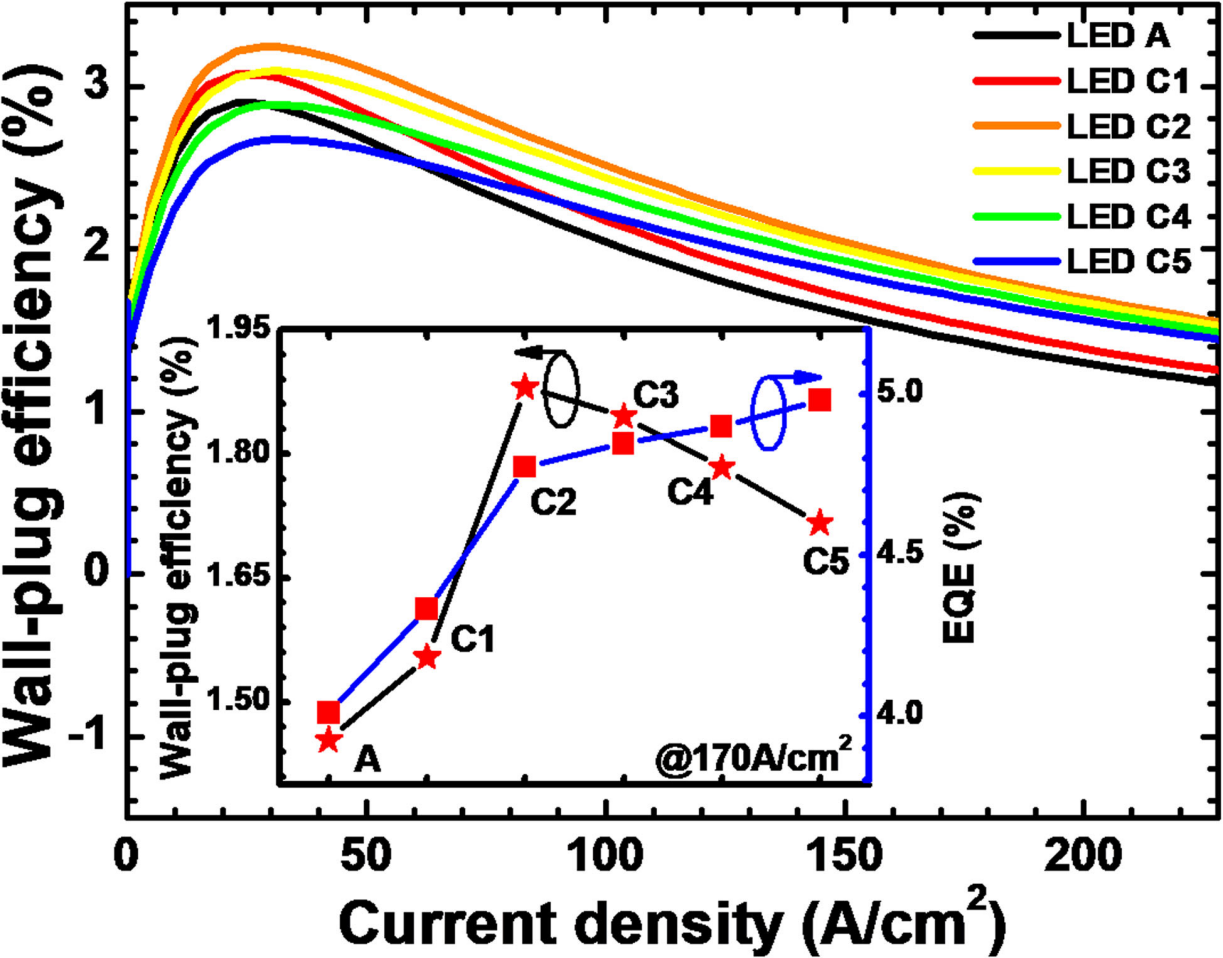
Relationship between WPE and the injection current for LEDs A and C*i* (*i* = 1, 2, 3, 4, and 5). Inset: the EQE and WPE for the investigated LEDs structured with a p-AlGaN layer with various AlN components when the current density is 170 A/cm^2^

The WPE as a function of the injection current density for all studied devices is exhibited in Fig. 8. When compared to LED A, the WPE of LED C1 increases once the NPN-AlGaN junction is adopted. WPE for LEDs C*i* (*i* = 2, 3, 4, and 5) can be further improved when the AlN composition of the p-AlGaN layer increases for the NPN-AlGaN junction. However, LED C2 shows the highest WPE owing to the lowest forward operating voltage despite the relatively low optical power density among LEDs C*i* (*i* = 2, 3, 4, and 5). In addition, we show the WPE and EQE at the injection current density of 170 A/cm^2^ for all investigated devices in the inset figure. It is well known that the current crowding effect is serious at a high injection current density. The NPN-AlGaN junction for LED C5 plays best in homogenizing the current. However, the WPE is not satisfactory once the forward operating voltage significantly increases. Therefore, one shall fully optimize the value of AlN component of the p-AlGaN insertion layer for the NPN-AlGaN junction before one can get the enhancement for both EQE and WPE.

### Effect of the Mg Doping Concentration for p-AlGaN Layer on the Current Spreading Effect

**Fig. 9:**
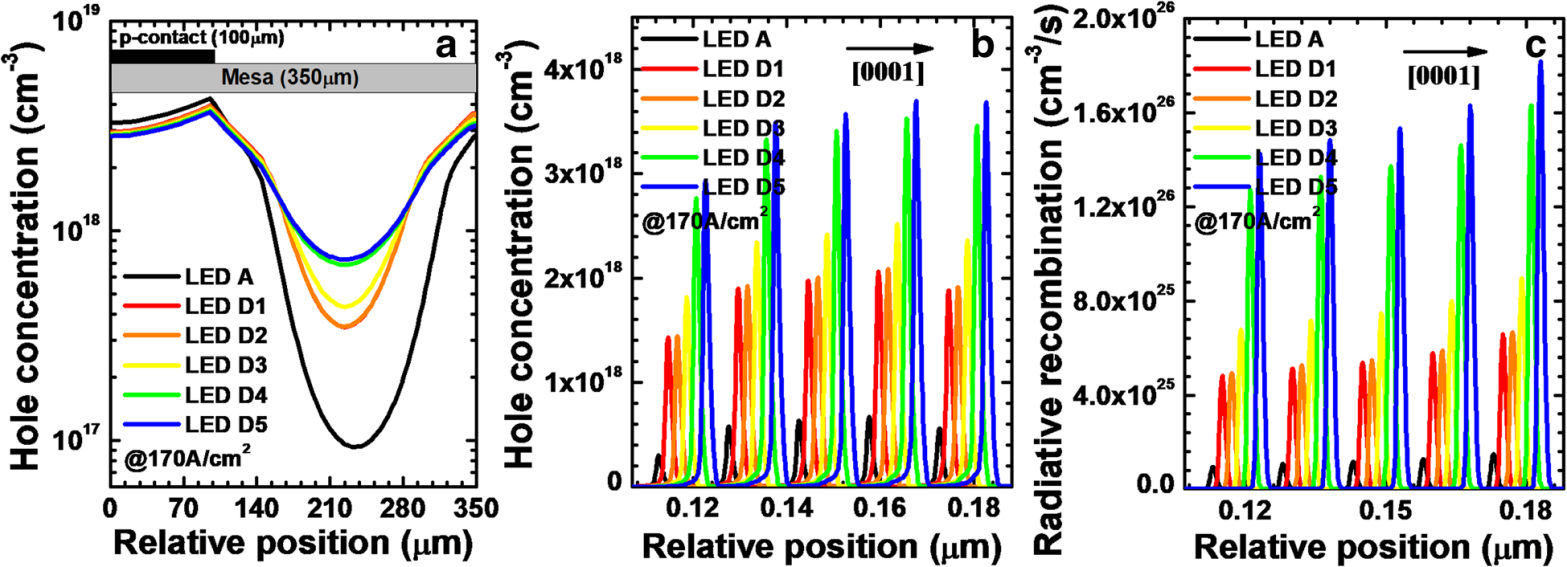
**a** Horizontal hole concentration in the LQW, **b** hole concentration levels, and **c** radiative recombination profiles in the MQWs for LEDs A and D*i* (*i* = 1, 2, 3, 4, and 5) when the current density is 170 A/cm^2^. We purposely shift the curves for **b** and **c** by 2 nm for easier identification

The width of the depletion region for the NPN-AlGaN junction can be managed by varying the Mg doping concentration for the p-AlGaN insertion layer, and the conduction band barrier height will also change accordingly. Thus, the value of *R*_*npn*_ can be increased once the depletion region for NPN-AlGaN junction becomes wide, and the value of *I*_1_/*I*_2_ will be reduced, i.e., the current spreading effect for DUV LEDs can be improved. For better elucidating the point, five DUV LEDs with different Mg doping concentrations for the p-AlGaN insertion layer in the NPN-AlGaN junction have been designed and investigated. We set the Mg doping concentrations for the p-AlGaN layer to 3 × 10^17^, 7.5 × 10^17^, 1.7 × 10^18^, 2 × 10^18^, and 3 × 10^18^ cm^−3^ for LEDs D*i* (*i* = 1, 2, 3, 4, and 5), respectively. The thickness and the AlN composition for the p-AlGaN insertion layer are 20 nm and 0.61, respectively. We adopt two NPN-AlGaN junctions. As shown in Table 2, the conduction band barrier height becomes increased as the Mg doping concentration increases for the p-AlGaN layer. Then, we calculate and show the lateral hole concentration in the last quantum well when the current density is 170 A/cm^2^ in Fig. 9a, and it is obvious that, compared with the lateral hole distribution for LED A, the lateral hole distribution becomes more uniform when the NPN-AlGaN junction is introduced for DUV LEDs. Moreover, even more homogenized hole distribution can be obtained once the Mg doping concentration for the p-AlGaN layer in the NPN-AlGaN junction increases.

**Table 2:** Conduction band barrier heights of each NPN-AlGaN junction for LEDs D*i* (*i* = 1, 2, 3, 4, and 5)

LEDs	D1	D2	D3	D4	D5
*φ*_1_ (eV)	0.078	0.117	0.234	0.254	0.267
*φ*_2_ (eV)	0.078	0.117	0.238	0.254	0.268

Then, the calculated hole concentration levels and radiative recombination profiles in the MQWs are demonstrated for all studied LEDs in Fig. 9b and c when the current density is 170 A/cm^2^, respectively, and the location where the data are collected is 120 μm away from the right mesa edge. As expected, LEDs D*i* (*i* = 1, 2, 3, 4, and 5) have the higher hole concentration levels and radiative recombination profiles in the MQWs when compared to LED A, while the hole concentration and radiative recombination increase with the increasing Mg doping concentrations in p-AlGaN layer for the LEDs with NPN-AlGaN junctions. We contribute the rising hole concentration in the MQWs for LEDs D*i* (*i* = 1, 2, 3, 4, and 5) to the enhanced current spreading effect.

**Fig. 10:**
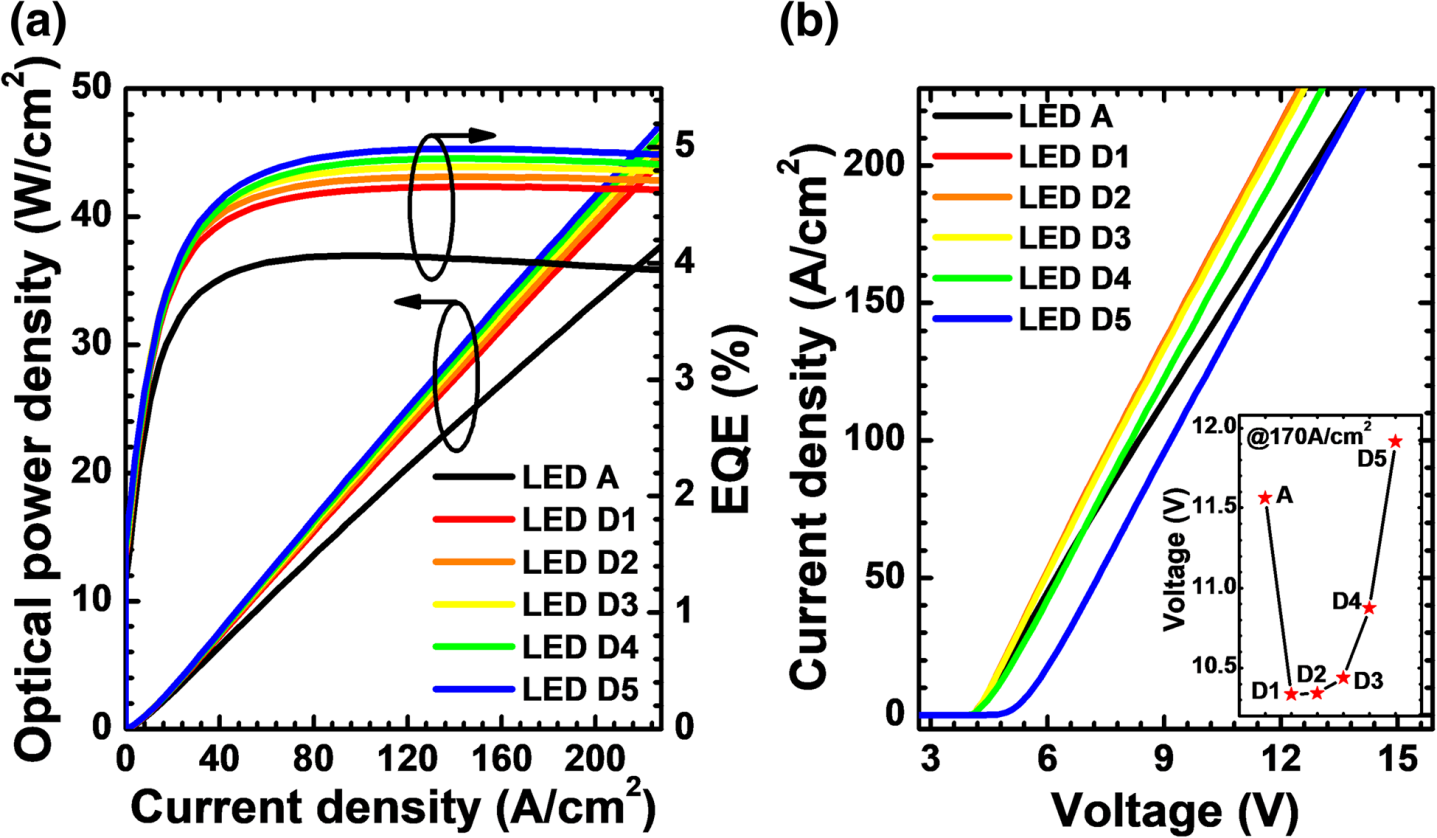
**a** EQE and optical power density in terms of the injection current and **b** current-voltage characteristics for LEDs A and D*i* (*i* = 1, 2, 3, 4, and 5). Inset: the forward voltages for LEDs A and D*i* (*i* = 1, 2, 3, 4, and 5) when the current density is 170 A/cm^2^

Owing to the reduced current crowding effect and the rising hole concentration in the MQWs, LEDs D*i* (*i* = 1, 2, 3, 4, and 5) accordingly show the promoted EQE and optical power density (see in Fig. 10a). The current-voltage characteristics for LEDs A and D*i* (*i* = 1, 2, 3, 4, and 5) are illustrated in Fig. 10b. Apparently, the forward operating voltages for LEDs D*i* (*i* = 1, 2, 3, 4, and 5) increase with the increasing Mg doping concentration for the p-AlGaN insertion layer. Among them, LED D5 shows the largest turn-on voltage, and this is ascribed to the parasitic diode that is caused by the very high level of Mg doping concentration in the p-AlGaN layer. According to the inset figure of Fig. 10b, it is also seen that LED D5 shows the largest forward operating voltage among all studied LEDs when the injection current density is 170 A/cm^2^.

**Fig. 11:**
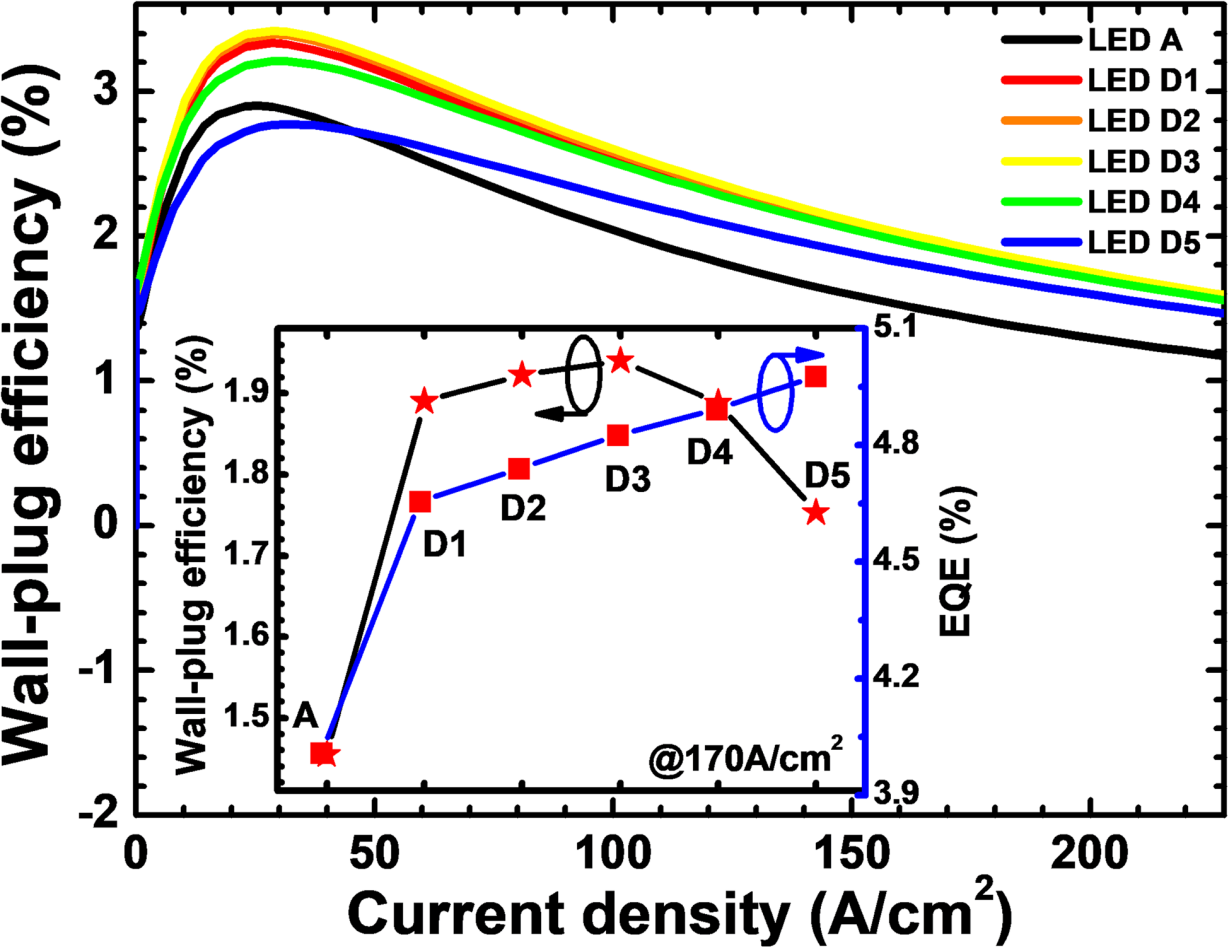
Relationship between WPE and the injection current for LEDs A and D*i* (*i* = 1, 2, 3, 4, and 5). Inset: the EQE and WPE for the investigated LEDs structured with a p-AlGaN layer with various doping concentrations when the current density is 170 A/cm^2^

For a more comprehensive analysis, we calculate the WPE as a function of the injection current density for all studied LEDs as shown in Fig. 11. The WPEs for LEDs D*i* (*i* = 1, 2, 3, and 4) are higher than that for LED A. The WPE for LED D5 exceeds that for LED A only when the injection current density is larger than 43 A/cm^2^. The lower WPE for LED D5 at the current density smaller than 43 A/cm^2^ is owing to the additional forward voltage consumption at the NPN-AlGaN junction as mentioned previously. From the inset figure, it can be seen that the EQE shows an ascending trend with the increase of Mg doping concentration for the p-AlGaN layer. However, the WPE decreases with the further increase of the Mg doping concentration for the p-AlGaN layer. Hence, we conclude that the current spreading effect and the forward voltage are very sensitive to the Mg doping level of the p-AlGaN insertion layer.

### Effect of the Thickness for p-AlGaN Layer on the Current Spreading Effect

In this section, the impact of the thickness for the p-AlGaN insertion layer in the NPN-AlGaN junction on the LED performance is investigated. First of all, two NPN-AlGaN junctions (i.e., NPNPN-AlGaN junction) are applied for all studied DUV LEDs, of which the AlN composition and the doping concentration for the p-AlGaN layer in the NPN-AlGaN junction are 0.61 and 1.5 × 10^18^ cm^−3^, respectively. We then set different thicknesses of 18, 20, 24, 28, and 32 nm for the p-AlGaN layer in LEDs T*i* (*i* = 1, 2, 3, 4, and 5), respectively. The calculated conduction band barrier heights for each NPN-AlGaN junction are presented in Table 3. It can be seen that the conduction band barrier height increases when the p-AlGaN layer in the NPN-AlGaN junction becomes thick, which enables the reduction of *I*_1_/*I*_2_ and correspondingly the improved current spreading.

**Table 3:** Conduction band barrier heights of each NPN-AlGaN junction for LEDs T*i* (*i* = 1, 2, 3, 4, and 5)

LEDs	T1	T2	T3	T4	T5
*φ*_1_ (eV)	0.117	0.210	0.250	0.255	0.258
*φ*_2_ (eV)	0.180	0.214	0.251	0.256	0.258

**Fig. 12:**
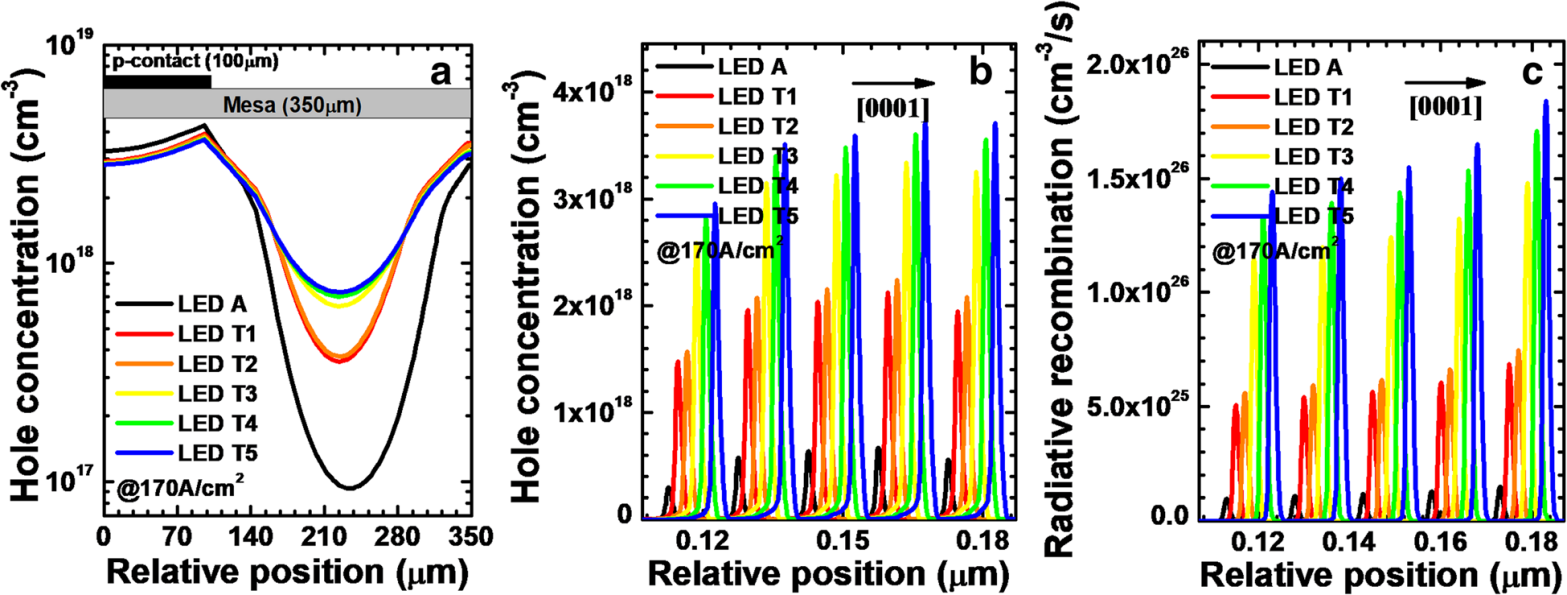
**a** Horizontal hole concentration in the LQW, **b** hole concentration levels, and **c** radiative recombination profiles in the MQWs for LEDs A and T*i* (*i* = 1, 2, 3, 4, and 5) when the current density is 170 A/cm^2^. We purposely shift the curves for **b** and **c** by 2 nm for easier identification

We calculate and show the horizontal hole concentration in the LQW for LEDs A and T*i* (*i* = 1, 2, 3, 4, and 5) when the current density is 170 A/cm^2^ in Fig. 12a. Clearly, the hole distribution becomes more homogeneous when the NPN-AlGaN junction is introduced in the DUV LED structure, and it becomes more uniform if the thickness for the p-AlGaN layer in the NPN-AlGaN junction gets larger. The reduced current crowding effect is ascribed to the higher conduction band barrier height in the depletion region caused by the thickened p-AlGaN layer in the NPN-AlGaN junction. Figure 12b and c exhibit the hole concentration levels and radiative recombination profiles, respectively, for LEDs A and T*i* (*i* = 1, 2, 3, 4, and 5) at the injection current density of 170 A/cm^2^. The hole concentration levels and radiative recombination profiles are collected at the location of 120 μm away from the right-hand edge of the mesa. We can see that, when compared to that of LED A in the MQWs, LEDs T*i* (*i* = 1, 2, 3, 4, and 5) show the higher hole concentration levels and thus higher radiative recombination profiles. Once the thickness of the p-AlGaN layer is increased, further enhanced hole concentration and radiative recombination in the MQWs can be obtained.

**Fig. 13:**
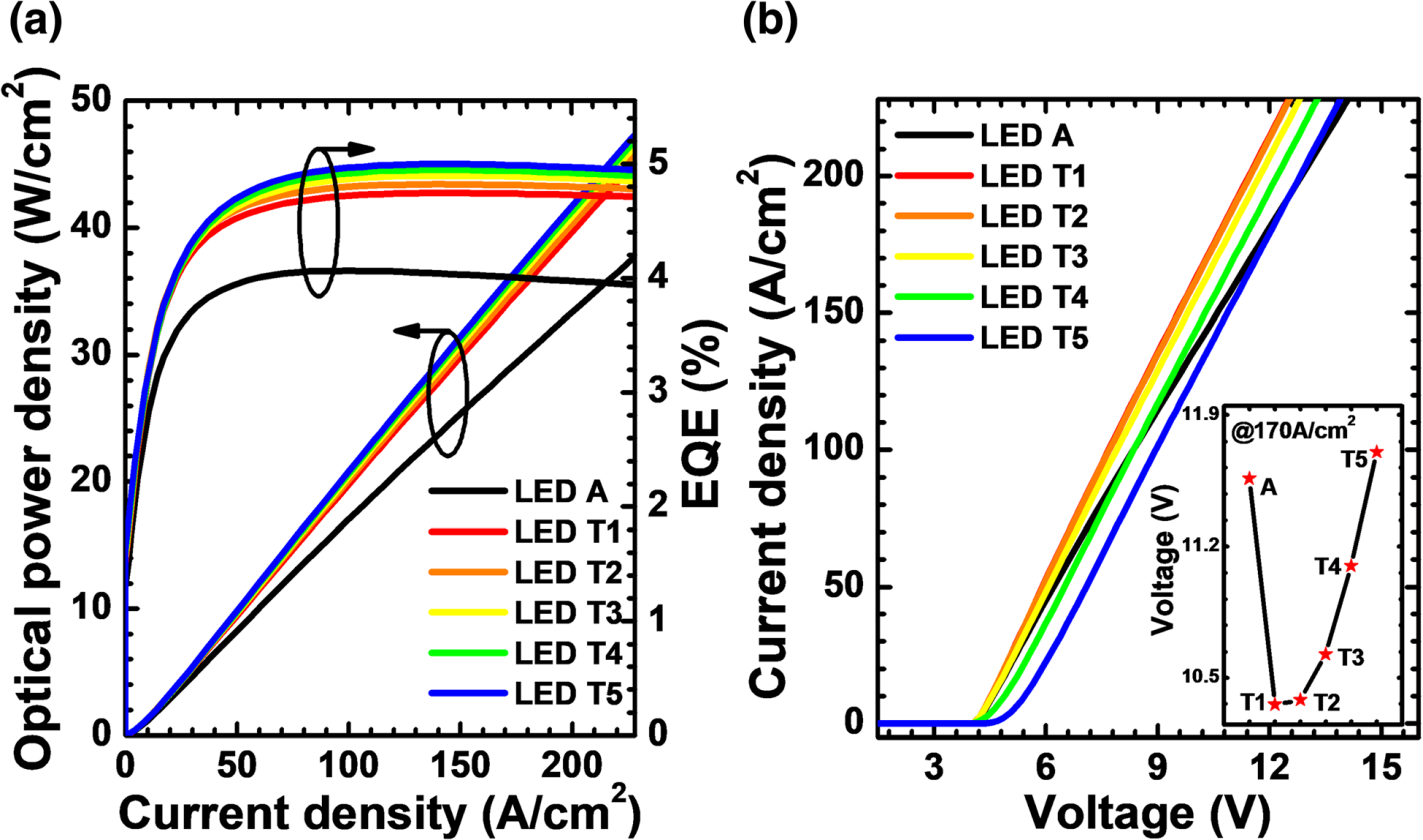
**a** EQE and optical power density in terms of the injection current and **b** current-voltage characteristics for LEDs A and T*i* (*i* = 1, 2, 3, 4, and 5). Inset: the forward voltages for LEDs A and T*i* (*i* = 1, 2, 3, 4, and 5) when the current density is 170 A/cm^2^

The observed optical power density and EQE for all studied LEDs in Fig. 13a agree well with the results shown in Fig. 12c, such that the increasing thickness for the p-AlGaN layer in the NPN-AlGaN junction can improve the optical power density and EQE. Moreover, we calculate and show the current-voltage characteristics for LEDs A and T*i* (*i* = 1, 2, 3, 4, and 5) in Fig. 13b. It shows that the forward operating voltages for LEDs T*i* (*i* = 1, 2, 3, and 4) exhibit a significant reduction when compared to that for LED A at the injection current density larger than 102 A/cm^2^, which is due to the significantly improved current spreading effect after adopting the NPN-Al_0.61_Ga_0.39_N junction as mentioned previously. However, a too thick p-AlGaN layer can cause an increase in the turn-on voltage owing to the parasitic N-AlGaN/P-AlGaN diode, e.g., LED T5 has the highest forward operating voltage among all the investigated LEDs when the current density is 170 A/cm^2^, which is also shown in the inset figure of Fig. 13b.

**Fig. 14:**
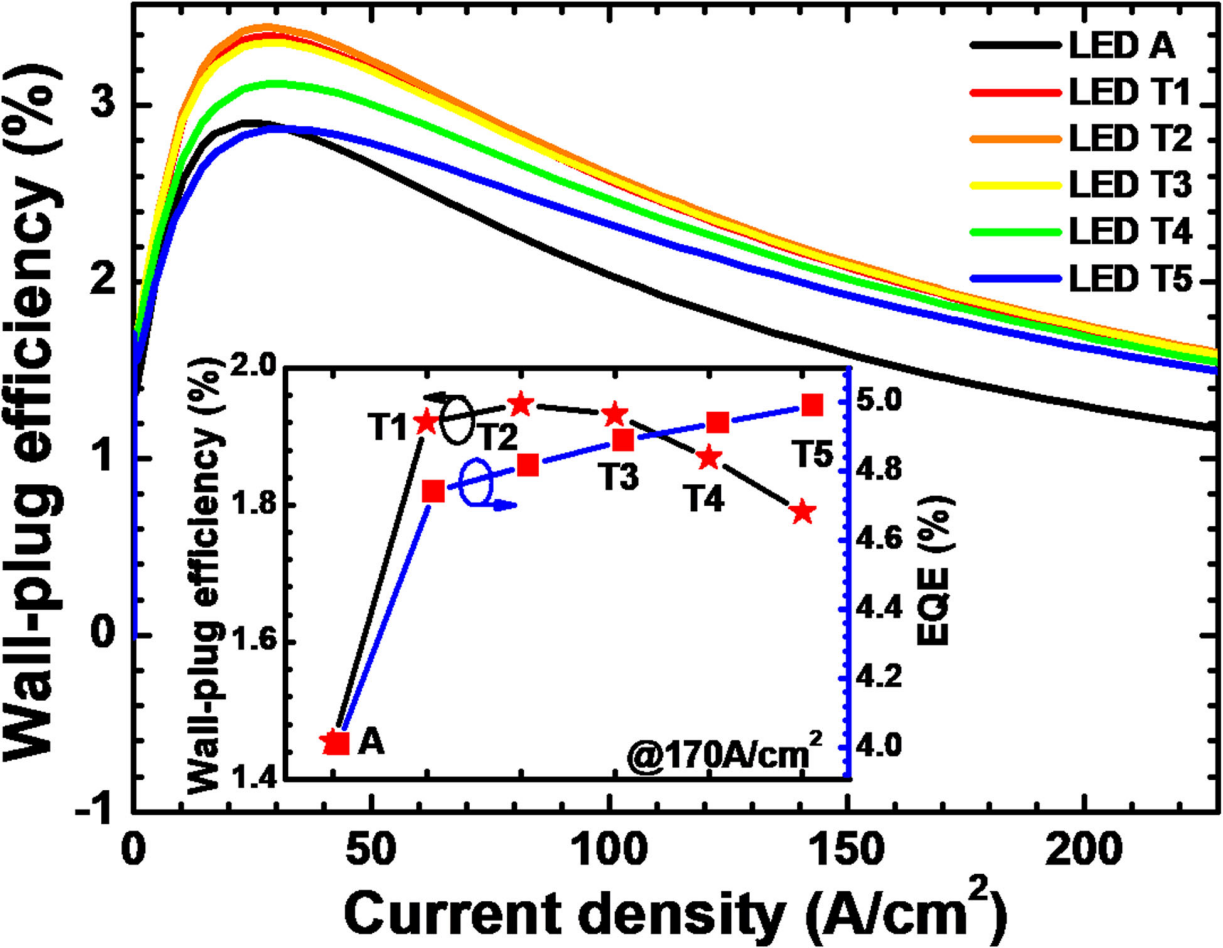
Relationship between WPE and the injection current for LEDs A and T*i* (*i* = 1, 2, 3, 4, and 5). Inset: the EQE and WPE for the investigated LEDs structured with a p-AlGaN layer with various thicknesses when the current density is 170 A/cm^2^

To this end, it is particularly important to further discuss the impact of higher forward operating voltage on DUV LED performance. Therefore, we calculate the WPE for all investigate devices and show the results in Fig. 14. We can see that the WPE for all LEDs with NPN-AlGaN junction exhibits distinct enhancement when compared to that for LED A. The presented WPEs in the inset figure also indicate that the NPN-AlGaN-structured DUV LED can save more electrical power than LED A. It is worth mentioning that the thickness for the p-AlGaN layer cannot be improved blindly, such that only when the thickness is properly set, then fully maximized WPE can be obtained.

### Effect of the NPN-AlGaN Junction Number on the Current Spreading Effect

**Fig. 15:**
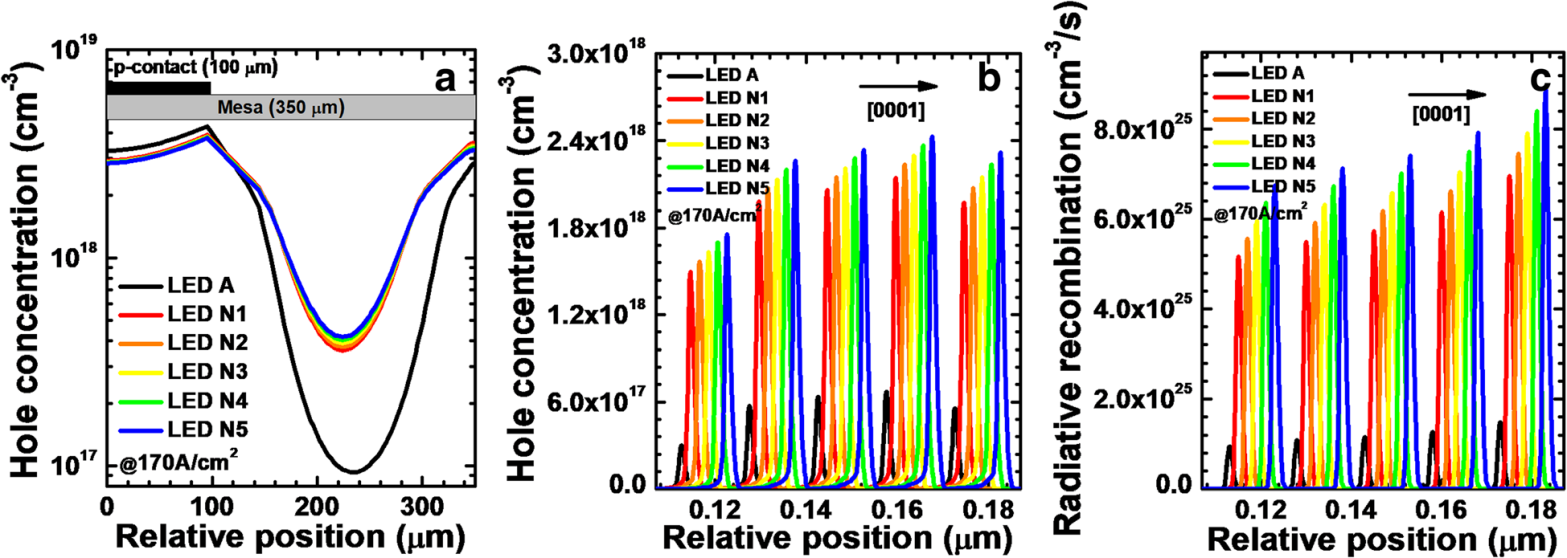
**a** Horizontal hole concentration in the LQW, **b** hole concentration, and **c** radiative recombination profiles in the MQWs for LEDs A and N*i* (*i* = 1, 2, 3, 4, and 5) when the current density is 170 A/cm^2^. We purposely shift the curves for **b** and **c** by 2 nm for easier identification

Finally, we investigate the influence of the NPN-AlGaN junction number on the current spreading effect. The p-Al_0.61_Ga_0.39_N layer is adopted in the NPN-AlGaN junction for the proposed DUV LEDs in this section, for which the Mg doping concentration and thickness are 1.5 × 10^18^ cm^−3^ and 20 nm, respectively. LEDs N*i* (*i* = 1, 2, 3, 4, and 5) have 1, 2, 3, 4, and 5 NPN-AlGaN junctions, respectively. As presented in Table 4, the conduction barrier heights of all NPN-AlGaN junctions are almost the same for LEDs N*i* (*i* = 1, 2, 3, 4, and 5). However, the total conduction barrier height for NPN-Al_0.61_Ga_0.39_N junctions in each investigated DUV LED surely increases when more NPN-Al_0.61_Ga_0.39_N junctions are utilized. Thus, the value of *N* × *R*_*npn*_ can be enhanced, which helps to better spread the current horizontally, i.e., the increased value of *I*_2_ in Eq. 3 is favored. The enhanced current spreading effect can be observed in Fig. 15a. The hole concentration in the LQW can become more uniform if the NPN-AlGaN junction number becomes more.

**Table 4:** Conduction band barrier heights of each NPN-AlGaN junction for LEDs N*i* (*i* = 1, 2, 3, 4, and 5)

LEDs	N1	N2	N3	N4	N5
*φ*_1_ (eV)	0.209	0.210	0.210	0.212	0.213
*φ*_2_ (eV)	–	0.214	0.214	0.214	0.214
*φ*_3_ (eV)	–	–	0.214	0.214	0.214
*φ*_4_ (eV)	–	–	–	0.214	0.214
*φ*_5_ (eV)	–	–	–	–	0.214

**Fig. 16:**
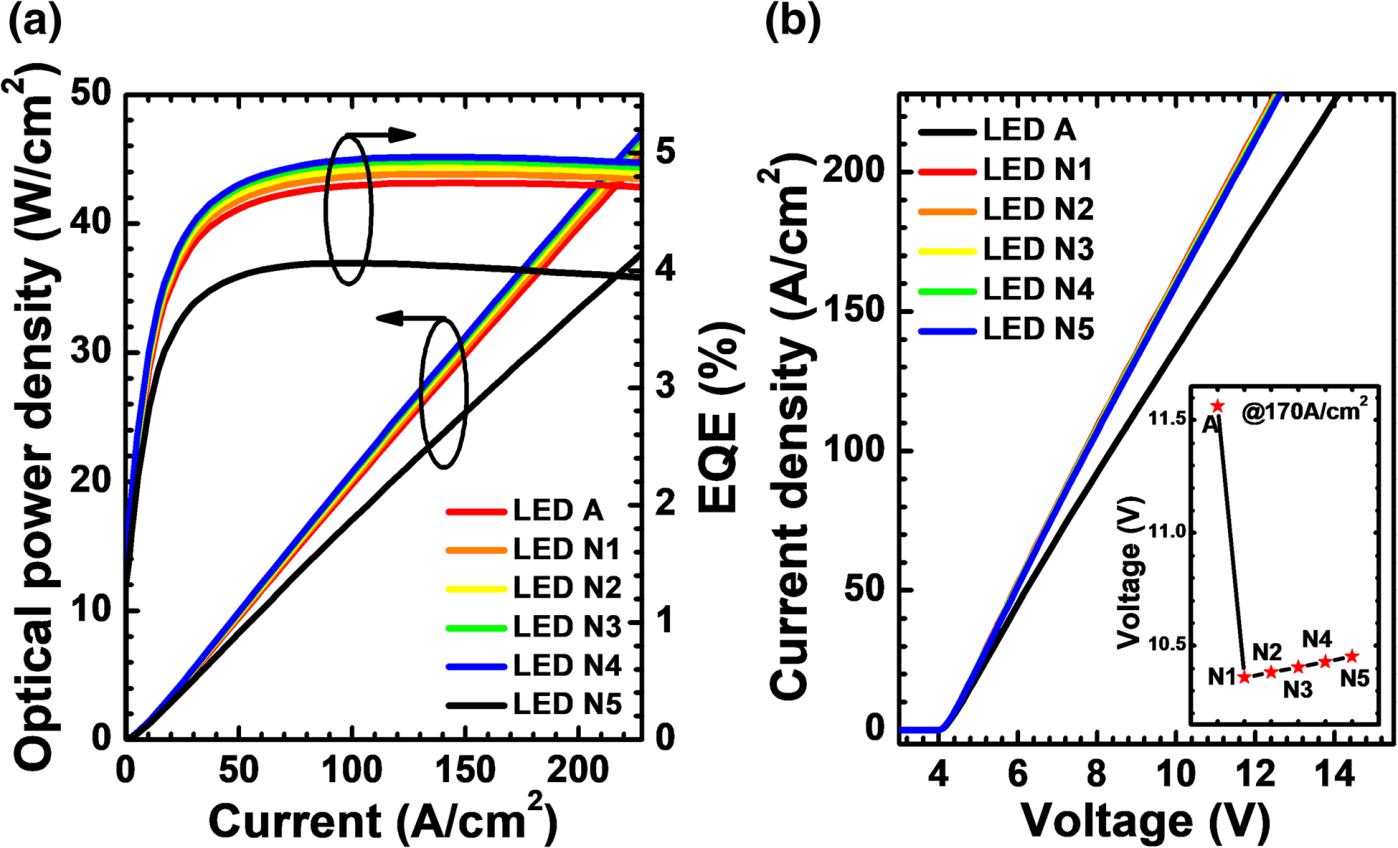
**a** EQE and optical power density in terms of the injection current and **b** current-voltage characteristics for LEDs A and N*i* (*i* = 1, 2, 3, 4, and 5). Inset: the forward voltages for LEDs A and N*i* (*i* = 1, 2, 3, 4, and 5) when the current density is 170 A/cm^2^

Then, the hole concentration levels and radiative recombination profiles in the MQWs for LEDs N*i* (*i* = 1, 2, 3, 4, and 5) when the current density is 170 A/cm^2^ are exhibited in Fig. 15b and c, respectively. We collect the hole concentration levels and radiative recombination profiles at the location of 120 μm away from the right-hand mesa edge. The hole concentration and radiative recombination are improved by using the NPN-Al_0.61_Ga_0.39_N junction, and further improvement can be obtained when more NPN-AlGaN junctions are included. Ascribed to the enhanced hole concentration in the MQWs, the optical power density and EQE for the DUV LEDs with NPN-AlGaN junction also shows a significant improvement. The current-voltage characteristics for all studied devices are shown in Fig. 16b, which illustrates that the forward operating voltages for LEDs N*i* (*i* = 1, 2, 3, 4, and 5) are lower than that for LED A, and this indicates that the current spreading effect can help to reduce the forward voltage once the Mg doping concentration, thickness, and AlN composition for the p-AlGaN layer are appropriately applied to the NPN-AlGaN junction. The turn-on voltage for all LEDs with NPN-AlGaN junction is almost the same as that for LED A, which illustrates the negligible impact of the reversely biased N-AlGaN/P-AlGaN parasitic junction if the Mg doping concentration in the p-AlGaN layer is properly set, i.e., the p-AlGaN layer has to be completely depleted before the device is biased.

**Fig. 17:**
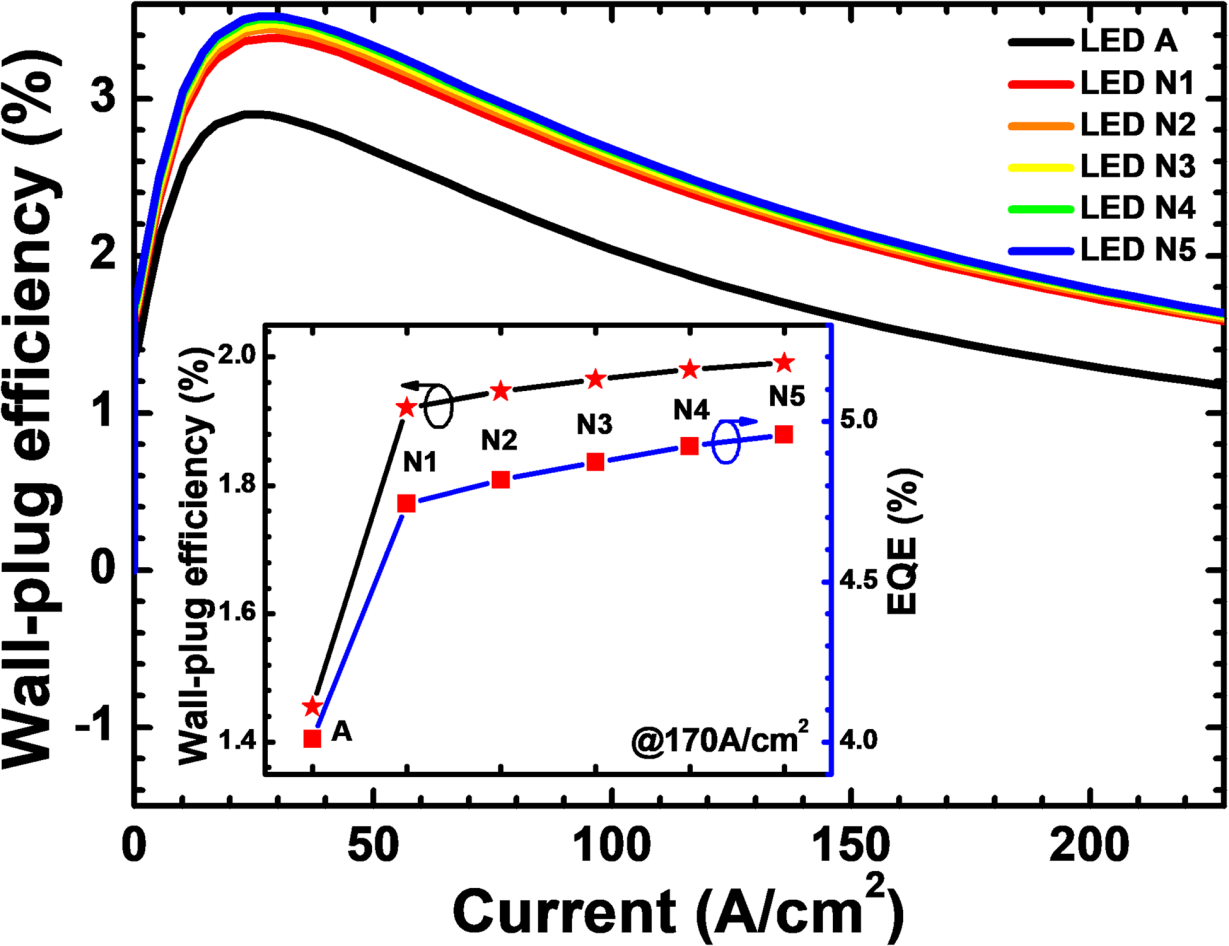
Relationship between WPE and the injection current for LEDs A and N*i* (*i* = 1, 2, 3, 4, and 5). Inset: the EQE and WPE for the investigated LEDs structured with various NPN-AlGaN number when the current density is 170 A/cm^2^

Last but not the least, the WPEs have also been demonstrated for LEDs N*i* (*i* = 1, 2, 3, 4, and 5) in Fig. 17. The WPEs of all DUV LEDs with NPN-Al_0.61_Ga_0.39_N junction have been promoted owing to the reduced forward operating voltage. In the inset figure, we show the EQE and WPE for LEDs A and N*i* (*i* = 1, 2, 3, 4, and 5) when the current density is 170 A/cm^2^. Although the EQE and WPE for LEDs N*i* (*i* = 1, 2, 3, 4, and 5) increase with the increasing of the NPN-AlGaN junction number, clearly, we can see that the magnitude of the increase is gradually decreasing, which indicates that the NPN-AlGaN junction number also shall be set to a proper number, and we firmly believe that the device will consume more electrical power if too many NPN-AlGaN junctions are adopted in DUV LEDs.

## Conclusions

To conclude, we have suggested embedding the NPN-AlGaN junction in the n-type electron supplier layer for DUV LEDs. After comprehensive and systematic discussions, we find that the NPN-AlGaN junction can reduce the current crowding effect in the p-type hole supplier layer and improve the hole injection for DUV LEDs. The NPN-AlGaN junction can tune the conductivity for the n-type electron supplier layer so that the current path in the p-type hole supplier layer can be manipulated. For further explorations, we have investigated the impact of different parameters for NPN-AlGaN junctions on the current spreading effect, the EQE, and the WPE. We find that the current can be further homogenized if the AlN composition, the Mg doping concentration, the thickness of the p-AlGaN insertion layer, and the NPN-AlGaN junction number are increased properly. Although the EQE can be promoted by using the proposed NPN-AlGaN junctions, the WPE is not always monotonically improving, which arises from the additional voltage drop at the barriers within the NPN-AlGaN junctions. Hence, more attention shall be made when designing NPN-AlGaN current spreading layers for DUV LEDs. However, we firmly believe that our results have provided an alternative design strategy to reduce the current crowding effect for DUV LEDs. Meanwhile, we also have introduced additional device physics and hence are very useful for the community.

## Data Availability

The data and the analysis in the current work are available from the corresponding authors on reasonable request.

## Abbreviations

APSYS: Advanced Physical Models of Semiconductor Devices
CL: Current spreading layer
DUV LEDs: Deep ultraviolet light-emitting diodes
EQE: External quantum efficiency
ITO: Indium tin oxide
LQW: Last quantum well
MQWs: Multiple quantum wells
NPN-AlGaN: n-AlGaN/p-AlGaN/n-AlGaN
IQE: Internal quantum efficiency
SRH: Shockley-Read-Hall
WPE: Wall-plug efficiency
ZGO: Zinc gallate
